# Update on Non-surgical Treatments for Lumbar Pain

**DOI:** 10.1055/s-0045-1810404

**Published:** 2025-11-04

**Authors:** André Wan Wen Tsai, Márcio Fin, Ibrahim Afrânio Willi Liu, Rosana Fontana, Sérgio Mendonça Melo Junior, Jose Eduardo Nogueira Forni

**Affiliations:** 1 Hospital das Clínicas da Faculdade de Medicina da Universidade de São Paulo, São Paulo, SP, Brazil; 2 Clínica Dorto, Sete Lagoas, MG, Brazil; 3 Pain Clinic, Hospital Madre Teresa, Belo Horizonte, MG, Brazil; 4 Plural Clínica da Dor, Novo Hamburgo, RS, Brazil; 5 Cure Centro Clínico, Morrinhos, GO, Brazil; 6 Faculdade de Medicina de São José do Rio Preto, São José do Rio Preto, SP, Brazil

**Keywords:** analgesia, conservative treatment, low back pain, multimodal treatment, musculoskeletal pain, analgesia, dor lombar, dor musculoesquelética, tratamento conservador, tratamento multimodal

## Abstract

Low back pain (LBP) is a very prevalent clinical condition worldwide. Approximately 90% of the cases are of nonspecific LBP, due to the lack of anatomopathological changes as potential causes of pain. A more assertive therapeutic approach requires identifying red or yellow flags and diagnosing the pain pattern (nociceptive, neuropathic, nociplastic, or mixed pain). Acute LBP prognosis is favorable in most cases, and the therapeutic objective is to prevent chronicity. For chronic LBP, the goals include pain reduction and improvements in functionality and quality of life. In acute and chronic cases, the principle of multimodal analgesia guides the treatment, which combines pharmacological, non-pharmacological, and interventional methods. The most common medications for LBP are simple analgesics, such as paracetamol, muscle relaxants, nonsteroidal antiinflammatory drugs (NSAIDs), and opioid analgesics. Opioids and NSAIDs should be prescribed at the lowest dose and for the shortest possible time. Patients with neuropathic components may receive adjuvant drugs. Phytocannabinoids may play a role when the previous pharmacological treatment fails. Physical methods, including heat, laser, and extracorporeal shock wave therapy, improve local circulation, produce muscle relaxation, and treat the myofascial component. Interventions such as acupuncture and radiofrequency promote peripheral and/or central neuromodulation. Aligning the patient's expectations to the outcomes of the proposed treatments is essential; to do so, we must consider the educational measures, behavioral therapies, and physical rehabilitation.

## Resumo

A dor lombar (DL) é condição clínica muito prevalente no mundo. Cerca de 90% dos casos são classificados como lombalgias inespecíficas, pois não há alterações anatomopatológicas que justifiquem sua dor. Para uma abordagem terapêutica mais assertiva, é preciso identificar as bandeiras vermelhas ou amarelas, bem como diagnosticar o padrão de dor (nociceptiva, neuropática, nociplástica ou mista) desses pacientes. Nas lombalgias agudas, a evolução natural é favorável na maioria das vezes, e o objetivo do tratamento é evitar a sua cronificação. Nos casos das lombalgias crônicas, visamos a diminuição da dor, e a melhora da funcionalidade e da qualidade de vida. Tanto nos casos agudos quanto crônicos, guiamos nossas condutas pelo princípio da analgesia multimodal, que combina medidas farmacológicas, não farmacológicas e intervencionistas contra a dor. Entre os medicamentos mais utilizados estão analgésicos simples, como o paracetamol, relaxantes musculares, anti-inflamatórios não esteroidais (AINEs) e os analgésicos opioides. Os AINEs e os opioides, quando indicados, devem ser prescritos na menor dose e pelo menor tempo possível. As medicações adjuvantes estão indicadas na presença do componente neuropático. Os fitocanabinoides são possibilidade quando as drogas anteriores falham. Medidas físicas, como calor, laser e terapia de ondas de choque extracorpórea, melhoram a circulação local, produzem relaxamento muscular e tratam o componente miofascial. Intervenções como acupuntura e radiofrequência promovem neuromodulação periférica e/ou central. Alinhar a expectativa do paciente aos resultados dos tratamentos propostos é fundamental; para isso, devemos considerar as medidas educacionais, terapias comportamentais e a reabilitação física.

## Introduction

Low back pain (LBP) is one of the most common reasons for patients to seek medical care worldwide, and it affects a significant portion of the working-age population. In addition, it is the most common cause of limited physical activity and absenteeism from work. 1 The prevalence of LBP in the American population ranges from 10 to 30%, and 65 to 80% of the population has LBP at some point in their lifetime. 1 In Brazil, the 1-year incidence of LBP in the adult population exceeds 50%. Meanwhile, the incidence of its chronic form ranges from 4.2 to 14.7%, depending on factors such as level of schooling, regional socioeconomic index, obesity, and sedentary lifestyle. 2

The identification of the precise etiology of LBP is rare; most cases are nonspecific, presenting no anatomopathological abnormalities, such as root compression or systemic disease. 3 Patients with signs and symptoms suggestive of infection, malignancy, fractures, and spinal cord or radicular compression meet the criteria for the so-called *red flags* for LBP, requiring a proper approach with laboratory and imaging tests. 4 In addition to the red flags, it is crucial to evaluate psychological and emotional factors, such as anxiety, depression, catastrophism, kinesiophobia, and professional and personal dissatisfaction, which are predictive factors for chronic LBP (yellow flags). 5

Moreover, it is essential to identify the different pathophysiological components involving pain (that is, nociceptive, neuropathic, and nociplastic elements), which often present as a mixed LBP picture. 6 7

Most LBP cases respond to simple measures and do not require additional tests. Patients should be encouraged to resume their activities as soon as the pain allows it. The principle of multimodal analgesia guides the therapeutic approach, including pharmacological, non-pharmacological (physical methods, education, and rehabilitation), and interventional treatments. 8

### Pharmacological Treatment

The LBP pharmacological treatment guidelines derive from studies 9 with severe limitations, including short-term trials and those involving heterogeneous populations. The most commonly-prescribed oral medications for LBP are paracetamol and nonsteroidal antiinflammatory drugs (NSAIDs), muscle relaxants, antidepressants, anticonvulsants, and opioids. 9 These medications have similar efficacy in reducing pain, but they cause different side effects. Regardless of the drug class, evidence 9 indicates good short-term analgesic efficacy and safety, but an unclear long-term efficacy.

Drug selection relies on LBP duration, from acute (< 6 weeks) to subacute (6–12 weeks) and chronic (> 12 weeks), and NSAIDs are prescribed in all stages. With the development of LBP chronicity, we observe a decrease in opioid use and increases in the use of antidepressant and antiepileptic medications 10 ( Table 1 ).

**Table 1:** Prevalence of pharmacological class indication based on the progression time of low back pain 10

N: 22	NSAIDs	Muscle relaxants	Opioids	Paracetamol	Antidepressants	Anticonvulsants
Acute	54.5%	27.2%	36.3%	22.7%	9.1%	4.5%
Subacute	50%	22.7%	18.2%	18.2%	9.1%	4.5%
Chronic	59.1%	22.7%	27.2%	18.2%	27.2%	13.6%
Unclassified	18.2%	NAS	13.6%	9.1%	4.5%	NAS

**Abbreviations:** N, number of guidelines; NAS, not assessed; NSAIDs, nonsteroidal anti-inflammatory drugs.

### NSAIDs and Simple Analgesic Agents

Evidence suggests a short course of paracetamol or NSAIDs for LBP treatment, avoiding prolonged treatment. 9 A systematic review 9 found no significant differences between paracetamol and NSAIDs in LBP relief. However, unlike NSAIDs, paracetamol does not increase the risk of myocardial infarction or gastrointestinal bleeding, and it is a safer option for patients predisposed to these conditions. 9

Although the literature supporting dipyrone (metamizole) for LBP treatment is scarce, this drug remains widely used in Brazil. However, Sociedade Brasileira de Reumatologia 11 (the Brazilian Society of Rheumatology) recommends dipyrone for LBP treatment.

In a systematic review and meta-analysis, Wewege et al. 12 reported that confidence in the evidence regarding pain intensity reduction with several NSAIDs and analgesics was low or very low. Drugs such as tolperisone, aceclofenac with tizanidine, and pregabalin have shown some efficacy, but with significant uncertainties. Additionally, the analysis 12 indicated that some medications, such as tramadol and its combinations with paracetamol, had an association with a moderately-increased risk of events.

### Muscle Relaxants

Muscle relaxants can belong to two groups: 9

- Antispastics: baclofen, tizanidine, dantrolene, and diazepam. There is no recommendation for their use in nonspecific LBP, and these drugs are often indicated to treat spasticity associated with central nervous system diseases, such as multiple sclerosis. It is better to avoid benzodiazepines due to their potential for addiction. 8 9
- Antispasmodics: cyclobenzaprine and carisoprodol. Although playing a role in the short-term treatment (2 weeks) for acute conditions, their prolonged use is not recommended. 8 9

### Antidepressants

Tricyclic antidepressants (amitriptyline and nortriptyline) have demonstrated a slight pain reduction in patients with chronic LBP. Selective serotonin reuptake inhibitors have not shown any efficacy superior to placebo for chronic pain. In contrast, serotonin-norepinephrine reuptake inhibitors (venlafaxine and duloxetine) have shown analgesic potential in selected conditions. 9 Tricyclic antidepressants, duloxetine, and venlafaxine are first-line treatments for neuropathic pain. 13

### Anticonvulsants

Gabapentin may be indicated for chronic LBP in selected patients, such as those with spinal stenosis, neurogenic claudication, and radicular pain, especially if the response to the initial treatments was unsatisfactory. Other anticonvulsants, such as carbamazepine, pregabalin, and lamotrigine, have no formal recommendations due to limited evidence. However, for neuropathic pain, gabapentin is the first-line treatment, while pregabalin is the second-line therapy. 13

**Fig. 1:**
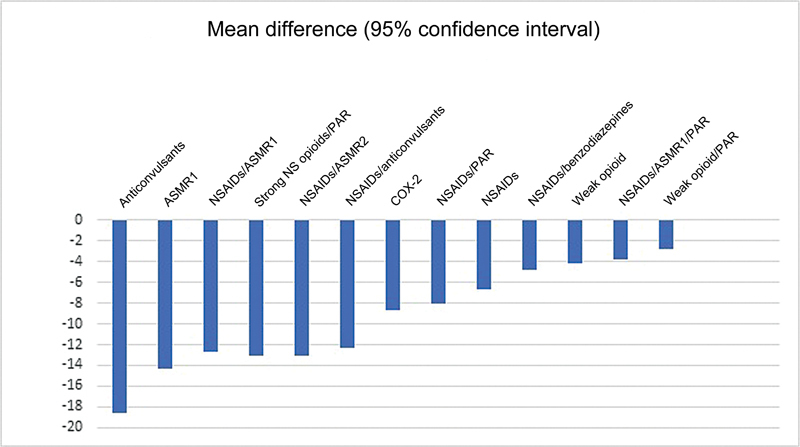
Comparison of analgesic effects of drug classes with placebo. Lines more to the right are more favorable to placebo interventions. **Abbreviations:** ASMR1, antispasmodic muscle relaxants; ASMR2, antispastic muscle relaxants; COX-2, cyclooxygenase-2 inhibitors; NS, non-selective; NSAIDs, nonsteroidal anti-inflammatory drugs; PAR, paracetamol. 12

Although a systematic review 12 indicated that anticonvulsants may cause moderate pain reduction compared to placebo, the evidence level is considered very low. Even so, they were superior to other therapeutic classes 12 ( Fig. 1 ).

### Opioids

Short-term opioid therapy may be indicated for the management of severe acute LBP, as supported by the literature. However, prolonged use requires care and strict supervision with the goals of patient rehabilitation and the achievement of well-defined therapeutic objectives. Treatment discontinuation should be considered in cases of therapeutic failure or repeated risk behavior, such as substance use disorder. 9 There is a trend towards a reduction in opioid prescriptions as LBP becomes chronic. 10

### Cannabis

Phytocannabinoids modulate pain pathways by activating cannabinoid receptors. Although the pharmacological mechanisms responsible for analgesic effects have not yet been fully understood, recent research has highlighted the anti-inflammatory properties of phytocannabinoids and their impact on pain relief. The literature on the effectiveness of cannabis as an analgesic is p wide. 14 A placebo-controlled review 15 concluded that there is moderate-quality evidence supporting the use of cannabinoids for chronic pain treatment.

Cannabinoid receptors are widely distributed in pain-modulating pathways, including peripheral and central sensory neurons, and they also influence the regulation of emotional responses to noxious and painful stimuli. At the supraspinal level, these receptors modulate the cognitive and emotional components of pain perception in the sensorimotor cortex and amygdala, reducing unpleasant pain-associated sensations. In the periphery, activation of type-1 cannabinoid receptors (CB1) inhibits the transmission of nociceptive stimuli, while activation of type-2 cannabinoid receptors (CB2) located in cutaneous immune cells hinders the release of inflammatory cytokines. 16

### Biological Agents

Animal model studies support the involvement of tumor necrosis factor alpha (TNF-α) in disc herniation, nerve sensitization, and ingrowth, demonstrating the preventive effects of blocking this inflammatory factor. Local TNF-α production is higher in herniated and degenerated discs, motivating clinical studies 17 to evaluate the efficacy of its inhibition in the treatment of discogenic pain in humans. Biological agents block or inhibit crucial steps in inflammation triggering and propagation, with a potential complementary role in LBP and sciatica management, especially when traditional interventions fail. However, despite promising results in preclinical studies, clinical evidence remains inconsistent. A study 17 tested two biological agent classes: anti-TNFs for patients with chronic LBP with radiculopathy and anti-nerve growth factor (NGF) for chronic non-radicular LBP. The quality of systematic reviews is low due to the heterogeneity of the studies. Furthermore, few studies have focused on the direct test of these therapies with other pharmacological options, and further research is required. Tanezumab may have greater effectiveness in pain control and functional improvements in chronic, nonspecific LBP. However, anti-NGF's inconsistent results do not support a recommendation for its use in LBP with no radiculopathy, as the evidence is weak. 17

### Physical Interventions

Heat and superficial cryotherapy increase blood circulation, followed by local cooling, potentially relieving muscle tension and improving joint movement restriction. These techniques demonstrate moderate short-term efficacy in reducing pain and disability in acute and subacute LBP, particularly when associated with physical exercise. However, most studies have limited evidence. For chronic LBP, the evidence on the efficacy of adjunctive thermotherapy (heat) and cryotherapy is insufficient and conflicting regarding the assessment of differences between them. 6 18

Therapeutic ultrasound (TUS) has not shown efficacy in LBP treatment. Low-quality evidence 19 failed to demonstrate differences between actual and sham ultrasound in reducing pain after treatment at a 4-week follow-up and compared with a control group. Furthermore, there were no significant effects on functional improvement. Evidence remains insufficient in the medium and long terms. 19

Evidence on transcutaneous electrical nerve stimulation (TENS) is also insufficient to prove efficacy in chronic LBP treatment. Studies with a low level of evidence 20 21 have not identified differences between actual and sham TENS in short-term pain intensity reduction or functional gain.

Low-level laser therapy (LLLT), or photobiomodulation, is the cutaneous application of different light wavelengths to promote biochemical reactions, decreasing local inflammation and pain. For LBP, low-quality evidence 22 23 suggests a small to moderate short-term improvement in pain and a small benefit in function.

In a meta-analysis, 22 high-level laser therapy (HILT) demonstrated a significantly better reduction in pain compared to the control group, in addition to improvements in the Oswestry and Roland-Morris' disability indices. A prospective clinical trial 23 comparing HILT and TUS revealed that HILT led to a statistically significant reduction in pain and disability after treatment and at a 3-month follow-up.

Extracorporeal shock wave therapy (ESWT) uses high-pressure, short-duration mechanical waves followed by a negative pressure stage. This process triggers a mechanotransduction phenomenon, resulting in biological effects on the affected tissue. These effects lead to pain reduction and increase local vascularization, tissue regeneration, and osteogenesis. Studies have indicated ESWT yields benefits for myofascial syndrome, contributing to extracellular matrix regulation. In a prospective randomized study, 24 ESWT and an exercise program decreased chronic LBP compared to exercise alone within 3 months; however, there was no significant impact on function.

The available evidence 19 was insufficient to prove the efficacy of other physical methods, such as electrical muscle stimulation and short-wave diathermy, in acute and subacute LBP and chronic pain, with or without radiculopathy.

### Interventional Methods

Interventional methods are medical procedures used for pain relief and/or diagnostic aid (block test), usually performed under imaging guidance. 25

In addition to the clinical treatments mentioned herein, specific interventions may be considered based on the structure involved in nonspecific LBP: 25

a) Discogenic component – more common in younger patients, often under 40 years of age, with exacerbated pain in trunk flexion;
b) Facet component – more frequent in elderly patients over the age of 60 presenting with pain in lumbar hyperextension;
c) Sacroiliac joint components – more prevalent in patients undergoing lumbar spine arthrodesis and presenting with worse pain when remaining seated;
d) Myofascial component – highly-prevalent in musculoskeletal pain, responding well to treatments such as acupuncture.

### Acupuncture

Acupuncture is a medical treatment widely used worldwide due to its proven clinical efficacy in several conditions, especially acute and chronic pain, including LBP. 19 26 27 Some mechanisms involved in acupuncture analgesia are well established, including reduced proinflammatory cytokine concentrations at the intervention point, modulation at the level of the posterior horn of the spinal cord, and activation of a descending inhibitory system affecting the serotonergic and the noradrenergic pathways. 27 In addition, the activation of the hypothalamic-pituitary-adrenal axis and the involvement of the endogenous opioid system are also well established. Other mechanisms remain under study, 27 including trigger point inactivation, the endocannabinoid system, mast cell degranulation, and transduction through A1, transient receptor potential cation channel subfamily V member 1 (TRPV1), and transient receptor potential cation channel subfamily V member 2 (TRPV2) adenosine receptors. Several studies 28 29 30 have demonstrated that acupuncture improves pain and function in LBP compared to the placebo group. Pain relief and improved function are observed, on average, after five sessions, and the best outcomes occur when acupuncture is associated with conventional treatment, whether pharmacological or not. 31

### Radiofrequency and Miscellaneous Blocks

Radiofrequency denervation is an invasive method for chronic nonspecific LBP treatment. Its indications include pain originating from a disc, a facet, or the sacroiliac joint. Although the outcomes are controversial, it is another tool for pain control. Despite the lack of robust scientific evidence on the duration of pain relief, leading experts 32 recommend it as an additional alternative in chronic LBP management.

The main interventions for LBP treatment include facet blocks, discogenic blocks, sacroiliac joint blocks, pulsed and ablative radiofrequency application in facets, and middle branch blocks.

For LBP with a discogenic origin, after diagnosis confirmation by physical examination, imaging tests, and provocative discography, the international literature 33 reports that treatment with intradiscal pulsed radiofrequency has led to good outcomes.

For LBP originating from facet joints, after clinical diagnosis, imaging tests, and confirmation by block test (in which an injection of 0.5 mL of 2% xylocaine into the facet leads to more than 70% of pain improvement), treatment can use joint ablative radiofrequency and pulsed radiofrequency in the medial branch of the spinal nerve. 34 35 36 Ablative radiofrequencies present better long-term results in pain control. 34 35 36

For pain originating from the sacroiliac joint, diagnostic confirmation includes clinical examination, imaging tests, and a block test with 0.5 mL 2% xylocaine injection. With a marked improvement in pain, treatment can use injectable dexamethasone with ropivacaine. 36 However, this procedure leads to short-term pain control. Alternatively, ablative radiofrequency or cryotherapy blockade of the sacroiliac joint may improve pain for a longer period. 37

### Education and Physical and Mental Rehabilitation

Education on musculoskeletal pain aims to provide relevant information about pain and the clinical condition, especially for patients with a higher degree of anxiety, worry, passive behavior, or fear. 38 Guiding patients to change their lifestyle for weight loss and smoking cessation and raising awareness of their role in the treatment increase the rate of good outcomes. 38

Cognitive-behavioral therapy (CBT) is an approach to chronic musculoskeletal pain with a small to moderate effect on chronic LBP improvement. The goal of CBT is to modify one of the three response systems (behavioral, cognitive, and physiological reactivity). The most promising interventions for physical treatment and rehabilitation in patients with chronic LBP are multidisciplinary treatment or behavioral treatment. All types of behavioral therapy were more effective in reducing pain intensity when compared with the control group. Furthermore, there is some evidence that the addition of behavioral components may decrease time off work and sick leave costs. 20 39

Another approach, mindfulness, seeks to reduce stress by encouraging the mind to focus on the present moment. Mindfulness may have a small to moderate short-term effect, but a lower medium-term effect over LBP. One study demonstrated that 60% of the patients undergoing this technique presented a 30% improvement compared to baseline values, with clinical significance after 6 months. When compared with the CBT group, 58% of the patients in the mindfulness group improved, as did 44% in the regular care group. 19 39 Both CBT and mindfulness aim to adjust thoughts and behaviors perpetuating pain. They have a moderate contribution to functional gain, which persisted for up to 2 years. 19 39

Exercise therapy also significantly reduced pain intensity and disability compared with regular care; the literature reports 20 40 strong evidence for strengthening exercises for the muscles stabilizing the spine and pelvis (the core muscles).

### Final Considerations

Low back pain is a highly-prevalent clinical condition in Brazil and worldwide. Specific causes, including fractures, herniated discs, canal stenosis, and systemic diseases, account for less than 10% of all cases. 10 19 Pharmacological treatment is part of the multimodal LBP management regardless of the clinical stage, with a preference for short-term use, especially of NSAIDs and opioids. Some drugs, such as gabapentinoids, tricyclic antidepressants, and dual serotonin and norepinephrine reuptake inhibitors, are the first option in cases of neuropathic pain with LBP. 13

Cannabis emerges as a treatment option when conventional medications fail. In addition, interventional methods for nonspecific LBP treatment represent an additional resource and an adjunct to other pharmacological modalities, aiming at faster and more effective rehabilitation.

Lastly, physical methods, education, and physical and mental rehabilitation for acute and chronic LBP patients play a crucial role.
